# Intense Intestinal Carriage of Carbapenemase-Producing Klebsiella pneumoniae Co-harboring OXA-48, KPC, VIM, and NDM Among Preterm Neonates in a Moroccan Neonatal Intensive Care Unit

**DOI:** 10.7759/cureus.50095

**Published:** 2023-12-07

**Authors:** Benboubker Moussa, Fouzia Hmami, Btissam Arhoun, Samira El Fakir, Abdelhamid M Massik, Salim Belchkar, Lahbib Hibaoui, Bouchra Oumokhtar

**Affiliations:** 1 Human Pathology Biomedicine and Environment Laboratory, Faculty of Medicine and Pharmacy, Sidi Mohammed Ben Abdellah University, Fez, MAR; 2 Neonatal Intensive Care Unit, University Hospital Hassan II, Faculty of Medicine and Pharmacy, Sidi Mohammed Ben Abdellah University, Fez, MAR; 3 Microbiology and Molecular Biology Laboratory, Faculty of Medicine and Pharmacy, Sidi Mohammed Ben Abdellah University, Fez, MAR; 4 Department of Epidemiology and Public Health, Faculty of Medicine and Pharmacy, Sidi Mohammed Ben Abdellah University, Fez, MAR; 5 Biomedical and Translational Research Laboratory, Faculty of Medicine and Pharmacy, Sidi Mohammed Ben Abdellah University, Fez, MAR; 6 Epidemiology and Health Science Research Laboratory, Faculty of Medicine and Pharmacy, Sidi Mohammed Ben Abdellah University, Fez, MAR; 7 Microbiology and Molecular Biology Laboratory, Microorganisms Team, Genomics and Oncogene Factors, Faculty of Medicine and Pharmacy, Sidi Mohammed Ben Abdellah University, Fez, MAR

**Keywords:** nicu, preterm infants, intestinal colonization, klebsiella pneumoniae, carbapenemase

## Abstract

Introduction: This study aimed to investigate the prevalence and the carbapenemase production ability of Klebsiella pneumoniae isolates from premature neonates’ intestinal tracts in a Moroccan neonatal intensive care unit

Methodology: Active rectal screening was performed among 339 preterm infants. The collected isolates were subjected to antibiotic susceptibility testing, phenotypic analysis of carbapenemase production, and molecular detection of carbapenemase genes.

Results: Out of 293 K. pneumoniae isolates collected, 31.05% (91) were resistant to carbapenem and produced carbapenemase, resulting in a 22.12% rate of intestinal carriage. Among the carbapenem-resistant K. pneumoniae isolates, 40.65% (37) had co-harbored carbapenemase genes. All isolates contained the blaOXA-48 gene, and the blaNDM, blaVIM, and blaKPC genes were detected in 30.76%, 9.89%, and 2.19% of the isolates, respectively. Out of 30.76% of these isolates had both the blaOXA-48 and blaNDM genes, 8.79% had both blaOXA-48 and blaVIM, and only 2.20% had both blaOXA-48 and blaKPC genes. Furthermore, 88.57% of carbapenem-resistantK. pneumoniae isolates co-harboring carbapenemase genes were genetically related strains.

Conclusions: This study revealed a high prevalence of intestinal carriage of carbapenem-resistant K. pneumoniae. Therefore, implementing effective screening and diagnostic measures, and focusing on antimicrobial stewardship are essential to preventing the spread of these resistant strains and minimizing the risk they pose to premature infants.

## Introduction

Carbapenemases have emerged as a significant threat to broad-spectrum lactam resistance in Enterobacteriaceae and other Gram-negative bacteria like Pseudomonas and Acinetobacter spp. This is attributable primarily to carbapenemases, enzymes encoded by genes found on mobile genetic elements [1]. Klebsiella pneumoniae, a member of the Enterobacteriaceae family, is one such species that has a high rate of resistance acquisition when compared to other bacteria in this family [2]. This pathogen is known to produce a variety of carbapenemases, including Ambler class A carbapenemases (e.g., KPC), Ambler class B metallo-b-lactamases (MBLs; e.g., NDM, IMP, VIM, etc.), and Ambler class D carbapenemases (e.g., OXA-48) [2,3]. The New Delhi metallo-b-lactamase (NDM) is the most common and dangerous because it imparts resistance to carbapenems and practically all hydrolyzable b-lactams and has swiftly spread worldwide [4].

Furthermore, K. pneumoniae is the primary cause of newborn sepsis in poor countries [5,6]. With rising resistance to all available b-lactam antibiotics for neonates (penicillin, monobactam, cephalosporins, and so on), the use of carbapenems has gradually increased, eventually leading to a global increase in carbapenem-resistant K. pneumoniae (CR-KP) in the last two decades [2] [7]. Often due to localized infection or colonization of the urinary, gastrointestinal, or respiratory tract that spreads through the bloodstream [8], It is resistant to various drugs at a minimal cost of adaptation, making it capable of generating epidemics in neonatal facilities. Previous studies have reported the presence of a single carbapenemase in K. pneumoniae, but in recent years, reports of the co-existence of multiple carbapenemases have been observed [9,10]; however, information regarding intestinal colonization in the preterm infant of such resistance is scarce.

Upon delivery, preterm neonates (born before 37 weeks of gestation) are a vulnerable population who frequently require long-term hospitalization. This is attributed to their general immaturity, underdeveloped mucosal surfaces, and the necessity for invasive interventions like central and peripheral venous lines, total parenteral nutrition, respiratory support, and prolonged stays within neonatal intensive care units (NICUs). Hospital environments, medical staff interaction, surfaces, and support equipment, lead to increased exposure to multidrug-resistant (MDR) bacteria, raising carbapenem-resistant enterobacteria (CRE) acquisition risk and transmission in this population [11].

Therefore, evaluating the carriage of CREs plays a critical role in mitigating cross-transmission risk. Patients who are colonized with CRE act as reservoirs of drug-resistant bacteria. In facilities that don't frequently conduct colonization screenings, transmission instances may remain undetected. It is crucial to evaluate the genetic resemblance among CRE isolates to understand the transmission patterns and formulate effective strategies to impede the dissemination of these bacteria [12].

In a previous study conducted at a neonatal care facility, we discovered a notable occurrence of colonization by MDR bacteria among preterm neonates within their first week of hospitalization. Nevertheless, we did not observe any CRE that co-harbored carbapenemase genes [13].

To date, there has been a lack of investigations into the presence of carbapenem-resistant K. pneumoniae in the intestines of preterm neonates in Morocco. Therefore, the objective of this study was to determine the prevalence of carbapenem-resistant K. pneumoniae, particularly those co-harboring carbapenemase genes in preterm infants' guts. Furthermore, we sought to examine the genetic mechanisms, strain similarity, and resistance evolution of K. pneumoniae by implementing active rectal screening during hospitalization.

## Materials and methods

Collection of clinical isolates

This is a prospective study that took place at the NICU department of a hospital in Morocco from November 2019 to November 2022. The study implemented a surveillance protocol consisting of fecal sampling at admission and later during hospitalization for all premature infants. The study collected 293 K. pneumoniae strains, which were identified based on their microscopic, morphologic, and biochemical characteristics.

In this study, 339 preterm infants were recruited. Written and oral informed consent was obtained from parents or legal guardians after explaining the purpose of the study. Babies who died or were discharged before 48 hours of hospitalization or those born after 37 weeks of pregnancy were excluded from the study, according to the World Health Organization's criteria.

The study was approved by the Ethics Committee of the Faculty of Medicine and Pharmacy and the Hassan II University Hospital of Fez, Morocco (approval CE.FMPF/38/22), and complied with the principles of the Ethics Declaration.

Screening and antimicrobial susceptibility

All collected isolates underwent antibiotic susceptibility testing using three carbapenem disks, and those that demonstrated resistance to at least one carbapenem including imipenem (10 µg), meropenem (10 µg), and ertapenem (10 µg) were identified as CR-KP and selected for further testing. The selected isolates were subjected to a panel of 14 antimicrobial disks, including amoxicillin/clavulanic acid (30 µg), ticarcillin/clavulanic acid (75 µg), piperacillin-tazobactam (30-6 µg), ceftazidime (30 µg), cefoxitin (30 µg), cefotaxime (30 µg), amikacin (30 µg), cefepime (30 µg), gentamicin (10 µg), levofloxacin (5 µg), ciprofloxacin (5 µg), nalidixic acid (30 µg), trimethoprim/sulfamethoxazole (25 µg) and fosfomycin (200 µg). The reference strain E. coli ATCC® 25922 was used as a control. The results were interpreted by referring to the European Committee on Antimicrobial Susceptibility.

Phenotypic detection of carbapenemase strains

NG-Test CARBA-5

NG-Test Carba 5 is an immunoassay designed to simplify the identification and differentiation of carbapenemase in clinical laboratories. This quick test, which takes less than 15 minutes, relies on the use of immunochromatography to detect the presence of the five most prevalent carbapenemase groups (KPC, OXA-48-like, VIM, IMP, and NDM) directly from bacterial colonies.

Modified Carbapenem Inactivation Method Test (mCIM)

The CLSI 2020 standard was employed for identifying carbapenemases in K. pneumoniae samples. The method involved placing a meropenem disk into a 2 mL mixture of tryptic soy broth (TSB) along with the specimen. Then, the mixture was incubated at 35°C. The disk was transferred to a Mueller-Hinton agar plate along with E. coli ATCC® 25922 and was incubated overnight. The zone of inhibition surrounding the disk was measured, and isolates with a zone diameter of 6-15 mm or pinpoint colonies within a 16-18 mm zone were identified as CPs (carbapenemase producers).

EDTA-Modified Carbapenem Inactivation Method (eCIM)

The combination of the eCIM test and mCIM was employed to identify the presence of MBLs in K. pneumoniae isolates. The eCIM procedure applies solely to Enterobacterales isolates that give positive results in the mCIM test. The execution and interpretation of this test followed the guidelines outlined in the CLSI 2020 recommendations.

Molecular analysis

Total DNA extraction was performed according to the boiling method described in the previous studies. To screen for β-lactamase-encoding genes in extended-spectrum beta-lactamases (ESBL)-producing K. pneumoniae strains, a 2 µL aliquot of the supernatant was used as a DNA template for polymerase chain reaction (PCR). The genes screened included blaCTX-M, blaTEM, blaSHV, OXA 48, KPC, blaVIM, IMP, and NDM. The amplification reactions were conducted in a 50 µl volume, and PCR products were detected on 1.5% agarose gels stained with ethidium bromide and visualized under UV light. Positive controls were used, including known β-lactamase-producing strains E. coli U2A1790, E. coli U2A1799, Salmonella sp. U2A2145, and Salmonella sp. U2A1446.

DNA Sequencing

To confirm the identification of the amplified products, both strands of the purified amplicons were sequenced using the same primers used for PCR amplification. The sequencing was done using a Genetic Analyzer, and the nucleotide and protein sequences were analyzed with NCBI software from the National Center for Biotechnology Information (NCBI) website (http://www.ncbi.nlm.nih.gov).

Enterobacterial Repetitive Intergenic Consensus-PCR (ERIC-PCR)

ERIC-PCR was conducted on K. pneumoniae clinical isolates following the procedure outlined by Doyle et al. [14] and the templates were used for PCR. The primers (Table 1) and ERIC-PCR conditions were applied according to the methodology described by Codjoe et al. [15]. The obtained ERIC fingerprinting data was transformed into a binary code, with '1' denoting the presence of a band and '0' indicating its absence. Subsequently, a dendrogram was constructed through unweighted pair-groups method using arithmetic averages (UPGMA) and Ward's hierarchical clustering algorithm, and a proximity matrix by Jaccard measure was generated to calculate the similarity index.

**Table 1:** Primers used in this study, expected PCR product sizes, and annealing temperatures (Ta). Notes: blaKPC, blaNDM, blaVIM, blaOXA-48, and blaIMP genes code for KPC, NDM, VIM, OXA-48-like, and IMP carbapenemases, respectively, blaCTX-M, blaSHV, and blaTEM genes code for extended-spectrum beta-lactamases (ESBLs). Pf, forward primer; Pr, reverse primer; Ta, annealing temperature; bp, base pair; PCR, polymerase chain reaction; ERIC-PCR, enterobacterial repetitive intergenic consensus PCR

PCR Reaction	Gene	Primer	Primer Sequence (5^,^ → 3^,^)	Expected PCR Product Size (bp)	Ta (◦C)	References
Monoplex	Bla_KPC_	P-_F_	CGTCTAGTTCTGCTGTCTTG	798	52°C	[16,14,17]
		P-_R_	CTTGTCATCCTTGTTAGGCG			
Monoplex	Bla_NDM_	P-_F_	GGTTTGGCGATCTGGTTTTC	621	52°C	
		P-_R_	CGGAATGGCTCATCACGATC			
Monoplex	bla_OXA-48_	P-_F_	GCGTGGTTAAGGATGAACAC	438	52°C	
		P-_R_	CATCAAGTTCAACCCAACCG			
Monoplex	Bla_VIM_	P-_F_	GATGGTGTTTGGTCGCATA	390	52°C	
		P-_R_	CGAATGCGCAGCACCAG			
Monoplex	Bla_IMP_	P-_F_	GGAATAGAGTGGCTTAAYTCTC	232	52°C	
		P-_R_	GGTTTAAYAAAACAACCACC			
Monoplex	bla_CTX-M 1_	P-_F_	GGTTAAAAAATCACTGCGTC	863	60 ◦C	
		P-_R_	TTGGTGACGATTTTAGCCGC			
Monoplex	bla_CTX-M 2_	P-_F_	ATGATGACTCAGAGCATTCG	865	60 ◦C	
		P-_R_	TGGGTTACGATTTTCGCCGC			
Monoplex	bla_CTX-M 9_	P-_F_	ATGGTGACAAAGAGAGTGCA	869	60 ◦C	
		P-_R_	CCCTTCGGCGATGATTCTC			
Monoplex	bla_SHV_	P-_F_	CGCCGGGTTATTCTTATTTGTCGC	795	60 ◦C	
		P-_R_	TCTTTCCGATGCCGCCGCCAGTCA			
Monoplex	Bla_TEM_	P-_F_	ATAAAATTCTTGAAGACGAAA	1079	52 ◦C	
		P-_R_	GACAGTTACCAATGCTTAATCA			
ERIC-PCR		P-_F_	AAGTAAGTGACTGGGGTGAGCG	Variable	45 ◦C	
		P-_R_	ATGTAAGCTCCTGGGGATTCAC			

Statistical analysis

Additional variables were created to perform statistical analysis. Frequency tables, crosstabs, dendrogram construction, similarity index calculation, and chi-square analysis were carried out using Statistical Package for the Social Sciences software SPSS® version 23 (IBM Corp., Armonk, NY, USA).

## Results

Population characteristics

Three hundred and thirteen nine preterm infants were prospectively recruited in this study. Among the population, 160 preterm infants (47.19%) were female, while 179 (52.80%) were male. Based on the hospitals’ records, the chronological age of gestation ranged from 26 weeks to 38 weeks, with 13 cases (3.83%) classified as extremely preterm, 60 cases (17.69%) as very preterm, and 266 cases (78.46%) as moderate and late preterm. The majority of 260 (76.69%) premature newborns had a birth weight ranging from 1000 to 2499 g, and 201 (59.29%) were delivered by vaginal birth (Table 2).

**Table 2:** Characteristics of preterm newborns infants according to the acquisition of CR-KP during hospitalization *IQR, Mean ± SD IQR, interquartile Range; SD, standard deviation; CR-KP, carbapenem-resistant Klebsiella pneumoniae

Characteristics	All preterm N=339	Preterm neonates with CR-KP		p-value
		Yes (75, 22.12 %)	No (264, 77.87%)	
Gender				
Male	180 (53.09)	39 (21.66)	141 (78.33)	0.829
Female	159 (46.90)	36 (22.64)	123 (77.35)	
Birth Weight				
Birth Weight (g) Mean*	IQR:1261-2249	2040.01±1168.33	1786.54±681.13	0.017
< 1000g	19 (5.60)	04 (21.05)	15 (78.94)	0.646
1000- 2499 g	260 (76.69)	55 (21.15)	205 (78.84)	
≥ 2500 g	60 (17.69)	16 (26.66)	44 (73.33)	
Delivery Mode				
Vaginal birth	201 (59.29)	47 (23.38)	154 (76.61)	0.500
Cesarean birth	138 (40.70)	28 (20.28)	110 (79.71)	
Prematurity				
Age of gestation Mean*	IQR:31-35	32.60±3.24	32.74±2.77	0.698
Moderate and late preterm (32 to 37 weeks)	232 (68.43)	52 (22.41)	180 (77.58)	0.952
Very preterm (28 to <32 weeks)	99 (29.20)	21 (21.21)	78 (78.78)	
Extremely preterm (less than 28 weeks)	08 (2.35)	‎02 (25.00)	‎06 (75.00)	
Length of stay				
Length of stay (Day)*	IQR:04-12	10.57±08.38	09.20±08.26	0.205
< 5 Day	110 (32.44)	17 (15.45)	93 (84.54)	0.040
≥ 5 Day	229 (67.55)	58 (25.32)	171 (74.67)	

Collection and antimicrobial susceptibility of the K.pneumoniae isolates

**Figure 1:**
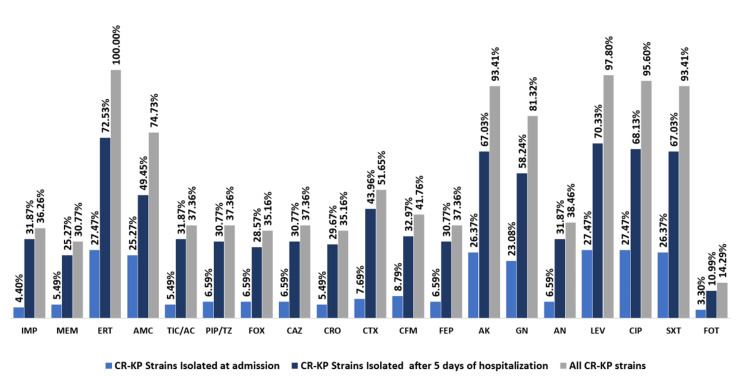
Antimicrobial resistance profile of 91 carbapenem-resistant Klebsiella pneumoniae strains to various tested antimicrobial agents IMP, imipenem; MEM, meropenem; ERT, ertapenem; PIP/TZ, piperacillin-tazobactam; TIC/AC, ticarcilline-acide clavulanique; AMC, amoxicilline-acide clavulanique; CAZ, ceftazidime; FEP, cefepime; CRO, ceftriaxone; CTX, cefotaxime; AK, amikacin; GN, gentamicin; CIP, ciprofloxacin; LEV, levofloxacin; NA, nalidixic acid; SXT, trimethoprim/sulfamethoxazole; FOT, fosfomycin; CR-KP, carbapenem-resistant Klebsiella pneumoniae

Following the screening protocol, a total of 293 non-duplicate K. pneumoniae isolates were recovered from 339 premature newborns through rectal screening. Out of these, 134 isolates (45.73%) were identified upon admission, while 159 isolates (54.26%) were detected after five days of hospitalization (Table 3). Among the 293 isolates of K. pneumoniae spp., a total of 91 isolates (31.05%) showed resistance to one or more tested carbapenems. These isolates were classified as CR-KP and were selected for further investigation, indicating a prevalence rate of 22.12% (75/339) for CR-KP intestinal carriage among the population recruited in our study. The antimicrobial susceptibility testing results confirmed that all 91 CR-KP isolates were MDR. They displayed the highest resistance rates to ertapenem (100%), ciprofloxacin (95.60%), levofloxacin (97.8%), ceftriaxone (84.37%) and ceftazidime (82.35%). On the other hand, the lowest rates of resistance were observed against imipenem (36.26%) and meropenem (24.17%), followed by fosfomycin (14.28%). Figure 1 illustrates the proportion of antimicrobial resistance among CR-KP isolates, while Table 3 presents their specific resistance patterns. Supplementary material (Supplementary Table 5) contains both the results of the antibiogram analysis for CR-KP isolates in this research and their corresponding identities to their genetic expression codes for ESBL, and carbapenemase enzyme production.

**Table 3:** Resistance patterns and phenotypic detection of carbapenemase production in Klebsiella pneumoniae isolates (n = 293) NG-Test Carba 5, rapid in vitro multiplex immunoassay for the phenotypic detection and differentiation of five common carbapenemase families (KPC, OXA-48-like, VIM, IMP, and NDM) EDTA, ethylenediaminetetraacetic

Antimicrobial Class	Antimicrobial Agents	Percentage of Resistance of Klebsiella Pneumoniae spp. isolates			P-value
		All Klebsiella Pneumoniae spp. isolates (n = 293)	Group of Klebsiella Pneumoniae spp isolates at admission (n=134)	Group of Klebsiella Pneumoniae spp isolates after 5 days of hospitalization (n=159)	
Carbapenems	Imipenem (10 µg)	33 (11.26 %)	04 (1.36 %)	29 (9.89 %)	p<0.05
	Meropenem (10 µg)	22 (7.50 %)	05 (1.70 %)	17 (5.80 %)	p<0.05
	Ertapenem (10 µg)	91 (31.05 %)	25 (8.53 %)	66 (22.52 %)	p<0.05
β-lactam combination agents	Piperacillin-tazobactam (30-6 µg)	185 (63.13%)	90 (30.71%)	95 (32.42%)	0.416
	Ticarcilline-acide clavulanique (75 µg)	120 (40.95%)	55 (18.77%)	65 (22.18 %)	0.747
	Amoxicilline-acide clavulanique (30 µg)	169 (57.67%)	81 (27.64 %)	88 (30.03 %)	0.676
Cephalosporins	Ceftazidime (30 µg)	216 (73.72%)	100 (34.12%)	116 (39.59%)	0.073
	Cefepime (30 µg)	182 (62.11%)	85 (29.01%)	97 (33.10%)	0.668
	Ceftriaxone (30 µg)	114 (38.90%)	53 (18.08%)	61 (20.81%)	0.612
	Cefoxitin (30 µg)	49 (16.72%)	15 (5.11%)	34 (11.60%)	0.034
	Cefotaxime (30 µg)	224 (76.45%)	102 (34.81%)	122 (41.63%)	0.987
Aminoglycosides	Amikacin (30 µg)	99 (33.78%)	30 (10.23%)	69 (23.54%)	p<0.05
	Gentamicin (10 µg)	274 (93.51%)	128 (43.68%)	146 (49.82%)	0.281
Fluoroquinolones	Ciprofloxacin (5 µg)	277 (94.53%)	127 (43.34%)	150 (51.19%)	0.946
	Levofloxacin (5 µg)	127 (94.91%)	46 (15.69%)	81 (27.64%)	p<0.05
	Nalidixic acid (30 µg)	73 (24.91%)	27 (9.21%)	46 (15.69%)	0.176
Folate pathway inhibitors	Trimethoprim/ sulfamethoxazole (25 µg)	274 (93.51%)	127 (43.34%)	147 (50.17%)	0.421
Fosfomycins	Fosfomycin (200 µg)	47 (16.04%)	23 (7.84%)	24 (8.19%)	0.630
Modified Carbapenem Inactivation Method (mCIM)		91 (31.05%)	25 (8.53%)	66 (22.52%)	p<0.05
EDTA-Modified Carbapenem Inactivation Method (eCIM)		13 (4.43%)	03 (0.68%)	11 (3.75%)	0.024
NG-CARBA 5 test		91 (31.05%)	25 (18.65%)	66 (41.50%)	p<0.05

Phenotypic detection of CR-KP

Carbapenemase enzyme production by CR-KP isolates (n = 91) was determined phenotypically using the NG-Carba 5 and mCIM tests. The eCIM test was used to detect MBL production, and the findings are reported in Table 3. Based on the NG-Carba 5 test findings, 91 (100%) K. pneumoniae isolates were carbapenemase-producing (CPs), and all strains were proven CPs by the mCIM test.

Extraction of DNA and amplification of ESBLs and carbapenemase genes

Out of the 91 CR-KP isolates, 37 (40.65%) were co-harbored carbapenemase genes. The DNA extracts were used as templates for PCR amplification of the carbapenemase genes blaKPC, blaNDM, blaVIM, blaOXA-48, and blaIMP. The PCR results revealed that the blaOXA-48 gene was detected in 91 (100.00%) of the tested isolates, while the blaNDM, blaVIM, and blaKPC genes were detected, respectively, in 28 (30.76%), nine (09.89%), and two (2.19%) of the CR-KP isolates. The PCR results showed that both blaOXA-48 and blaNDM genes were co-present in 28 (30.76%) isolates; blaOXA-48 and bla VIM were co-present in eight (08.79%); and blaOXA-48 and blaKPC genes were co-present in only two (2.20%) isolates. The presence of the blaIMP gene was not identified in the plasmid extracts. All CR-KP isolates also exhibited the presence of ESBL genes, although their distribution varied among OXA-48, NDM, and KPC isolates (Table 4).

**Table 4:** Genetic diversity of 91 carbapenem-resistant Klebsiella pneumoniae strains according to molecular detection of extended-spectrum b-lactamase and carbapenemase genes VIM, verona integron-encoded metallo-β-lactamase, NDM, New Delhi metallo-β-lactamase; KPC, Klebsiella pneumoniae carbapenemase; OXA-48, oxacillinase 48; CTX-M1, cefotaximase-Munich; SHV, sulfhydryl variable; TEM, temonera; CR-KP, carbapenem-resistant Klebsiella pneumoniae

	All CR-KP (n, %)	CR-KP strains Isolated at admission (n, %)	CR-KP strains Isolated after 5 days of hospitalization (n, %)
Carbapenemase genes			
OXA-48	91 (100%)	34 (37.36%)	57 (62.63%)
NDM	28 (30.76%)	05 (05.49%)	23 (25.27%)
VIM	09 (09.89%)	01 (01.09%)	08 (08.79%)
KPC	02 (02.19%)	-	02 (02.19%)
Combined carbapenemase genes			
OXA-48/NDM	28 (30.76%)	05 (05.49%)	23 (25.27%)
OXA-48/VIM	09 (09.89%)	01 (01.09%)	08 (08.79%)
OXA-48/KPC	02 (02.19%)		02 (02.19%)
Extended spectrum b-lactamase gene			
CTXM-1	79 (86.81%)	24 (26.37%)	51 (56.04%)
CTXM-2	10 (10.98%)	-	10 (10.98%)
CTXM-9	08 (08.79%)	02 (02.19%)	06 (06.59%)
SHV	48 (52.74%)	15 (16.48%)	33 (36.26%)
TEM	55 (60.43%)	16 (17.58%)	39 (42.85%)

Genotyping of CR-KP co-harboring carbapenemases-producing genes

**Figure 2:**
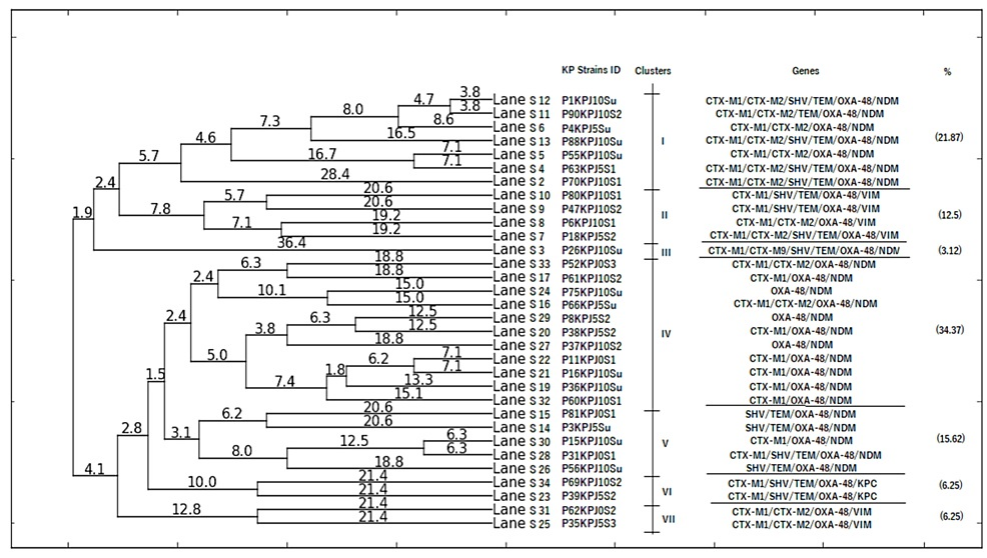
Dendrogram obtained by Dice coefficient for genetic dissimilarity between 32 CR-KP isolates co-harboring carbapenemase genes using ERIC-PCR, generated by PyElph 1.4 software with UPGMA analysis. VIM, verona integron-encoded metallo-β-lactamase, NDM, New Delhi metallo-β-lactamase; KPC, Klebsiella pneumoniae carbapenemase; OXA-48, oxacillinase 48; CTX-M1, cefotaximase-Munich; SHV, sulfhydryl variable; TEM, temonera; CR-KP, carbapenem-resistant Klebsiella pneumoniae; UPGMA, unweighted pair-group method with arithmetic mean; PCR, polymerase chain reaction; ERIC-PCR, enterobacterial repetitive intergenic consensus PCR

ERIC-PCR was performed on 32 K. pneumoniae isolates co-harboring carbapenemase genes to determine their genetic relatedness. The ERIC-PCR gel analysis exhibited a range from seven to 13 bands between the sizes of 100 and over 2000 bp (Supplementary Figure 3). As shown in Figure 2, the banding pattern observed with UPGMA analysis revealed that seven major clusters were formed (I to VII), illustrating genetic relationships among the isolates. The most frequent clusters were I (21.87%), II (12.5%), V (15.62%), IV (34.37%), VI and VII (06.25%), and Cluster III, which occurred in only one isolate. The dendrogram produced from the genomic DNA products of CR-KP isolates using ERIC-PCR indicates that the examined isolates have a common clonal origin. Based on the calculated Jaccard similarity index, isolates S25, S31, S23, and 34 were found to be 100% similar, as isolated S11 and S12, S5 and S4, S10 and S9, S7 and S8, S33 and S17, S24 and S16, S20 and S29, S22 and S21, S30, and S28, although they were all collected from different preterm babies. Apart from that, the remaining CR-KP isolates in this investigation displayed genetic dissimilarity, which was verified through the calculated similarity index.

## Discussion

The present study provided the first data on the prevalence and pattern associated with CR-KP intestinal colonization of preterm infants here in Morocco, focusing on tracking carbapenem-resistantK. pneumoniae during their NICU stay. This investigation involved active screening upon admission to the NICU and continued for over five days of hospitalization. Results indicated a CR-KP colonization rate of 22.12%, with 75 out of 339 preterm infants recruited in this study. Notably, this rate is higher compared to a similar neonatal intensive care unit in India, where the rate was 8.7% (26 out of 300 cases) [18]. Additionally, resistance to CR-KP increased with prolonged hospitalization, with 22.52% resistance cases after extended stays compared to 8.53% upon admission. As documented by various studies, this increase in CR-KP colonization prevalence correlates with prolonged hospitalization. In this way, Ruiz et al. also reported that the prevalence of colonization by multi-drug-resistant K. pneumoniae (MRKP) exceeded 50% in patients staying in the NICU for more than three weeks [18], suggesting that the hospital environment heightens the risk of colonization. Initial CR-KP carriage rates upon ICU admission varied widely globally, from under 1% in South Korea [19] to over 30% in Iran [20], and China also reported varying carriage frequencies between 6.5% [21] and 20.8% [22] for CR-KP through random screening during NICU stays.

The main carbapenemase genes found in this study were blaOXA-48 and blaNDM in strains of CR-KP. Furthermore, a majority of CR-KP strains were resistant to multiple antibiotics, and they showed similar characteristics of drug resistance. Other studies have found that blaNDM is the primary CR-KP gene found in neonatal patients [17], whereas blaKPC has been found in adults and older children [23]. Our findings also confirmed blaNDM as the prevailing carbapenemase gene within CR-KP isolates, aligning with prior research [17,24], and other reports from China [25]. Additionally, we identified two CR-KP strains expressing the blaKPC gene found in our study. In contrast to other carbapenemase genes, the blaKPC gene has higher abilities to colonize and transmit, resulting in more frequent cases of decolonization within a month (around 68%) in preterm neonates' intestinal colonization [26].

In this research, we successfully identified a prevalence of 47.7% in CR-KP co-harbored carbapenemase genes. This characterization revealed a coexistence of blaOXA-48 and blaNDM genes (30.76%), blaOXA-48 and blaVIM (08.79%), and blaOXA-48 and blaKPC (2.20%). Many studies, especially those focused on K. pneumoniae species, have demonstrated this occurrence, either in strains that are carried or strains that cause infections [27,28]. The study conducted by Flores et al. is highly significant, it found that out of 11 cases examined, 27% (3 out of 11) showed a simultaneous presence of KPC, OXA-48, and VIM, while 46% (5 out of 11) exhibited the co-occurrence of KPC and VIM [29]. The fact that CR-KP strains carry multiple carbapenemase genes together creates major difficulties for healthcare systems and public health. It amplifies antibiotic resistance, complicates diagnostics, and creates treatment defeat.

Linking to previous research, we also investigated the clonal relatedness of CR-KP strains that carry the carbapenemase gene using ERIC-PCR. We found that 88.57% (32 out of 35) of the 91 CR-KP bacteria were clonally related, indicating that the colonization of CR-KP strains may act as a source of MDR, suggesting that the origin and spread of MDR in NICU could be attributed to the colonization of CR-KP strains. Out of the CR-KP isolates that carried carbapenemase genes, 75% of those with the blaNDM gene were found in four clusters, and 18.75% of isolates with the blaVIM gene were found in two clusters, while isolates with blaOXA-48 showed more distinct clonality, with 100% distributed in seven clusters and singletons were detected in blaKPC isolates. A recent study highlighted a clonal solid connection between CR-KP strains. In the case of CP-KP-producing strains (N = 30) and OXA-48 (N = 37), seven and eight clusters were identified, respectively, which highlights K. pneumoniae clonal expansion [26]. Clonality has been fully documented in healthcare [30], even though infection control measures have intensified, implying the potential for the emergence of new strains.

In this study, we used conventional molecular methods to determine the rate of intestinal carriage of KP-CRs and characterized the genetic traits of these strains. Our findings raise concerns about the potential for severe infections among preterm neonates. However, it is important to acknowledge the limitations of this study and advocate for the use of advanced molecular techniques such as multilocus sequence typing (MLST), pulsed-field gel electrophoresis (PFGE), or next-generation sequencing (NGS) in future research to explore the diversity of strains and their resistance profiles. Additionally, our study did not extensively explore the risk factors associated with the acquisition of CR-KP.

## Conclusions

Intestinal colonization by K. pneumoniae may increase the prevalence of corresponding CR-KP in neonatal intensive care units during hospitalization. The co-occurrence of carbapenemase genes in CR-KP strains during intestinal carriage adds a layer of complexity to the challenges posed by antibiotic-resistant bacteria. Understanding the dynamics of this carriage, implementing effective screening and diagnostic measures, and focusing on antimicrobial stewardship is crucial to preventing the spread of these resistant strains and minimizing the risk they pose to premature infants.

## Figures and Tables

**Figure 3:**
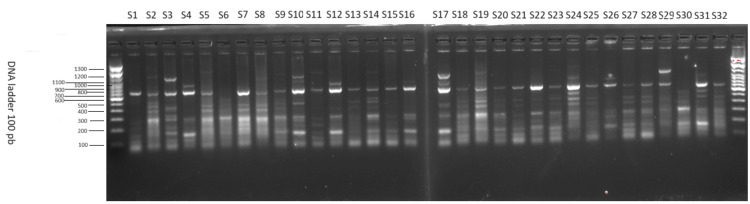
ERIC-PCR gel of 32 CR-KP isolates co-harboring carbapenemase plasmids ERIC-PCR, enterobacterial repetitive intergenic consensus PCR; CR-KP, carbapenem-resistant Klebsiella pneumoniae

## Appendices

**Table 5:** Antimicrobial resistance, carbapenemase enzyme production and genetic diversity among CR-KP isolates (n=91, (31.05%)) IMP, imipenem; MEM, meropenem; ERT, ertapenem; PIP/TZ, piperacillin-tazobactam; TIC/AC, ticarcilline-acide clavulanique; AMC, amoxicilline-acide clavulanique; CAZ, ceftazidime; FEP, cefepime; CRO, ceftriaxone; CTX, cefotaxime; AK, amikacin; GN, gentamicin; CIP, ciprofloxacin; LEV, levofloxacin; NA, nalidixic acid; SXT, trimethoprim/sulfamethoxazole; FOT, fosfomycin; CR-KP, carbapenem-resistant Klebsiella pneumoniae

CODE STRAIN	sample code	IMP	MEM	ETP	AMC	TIC/AC	PIP/TZ	FOX	CAZ	CRO	CTX	CFM	FEP	AK	GEN	AN	LEV	CIP	SXT	FOT	Ecim	Mcim	NG-Test® CARBA-5	blaCTX-M1	blaCTX-MNO	blaCTX-M9	blaSHV	blaTEM	blaOXA-48	blaNDM	blaVIM	blaKPC
P1KPJ10Su	J5	R	R	R	R	R	R	R	R	R	R	R	R	I	S	R	R	R	S	S	YES	YES	YES	YES	YES	NO	YES	YES	YES	YES	NO	NO
P3KPJ5Su	J5	R	I	R	R	I	I	R	R	I	R	I	I	S	S	I	R	R	S	S	NO	YES	YES	YES	YES	NO	NO	YES	YES	YES	NO	NO
P4KPJ5Su	J5	I	R	R	R	R	R	R	R	R	R	R	R	R	R	R	R	R	R	S	NO	YES	YES	YES	NO	NO	YES	YES	YES	NO	YES	NO
P6KPJ10S1	J5	R	R	R	S	R	R	R	I	R	R	R	R	R	R	R	R	R	R	S	YES	YES	YES	NO	NO	NO	YES	YES	YES	YES	NO	NO
P8KPJ5S2	J5	R	I	R	R	R	R	R	R	R	R	R	R	R	R	R	R	R	R	S	YES	YES	YES	YES	NO	NO	YES	YES	YES	YES	NO	NO
P11KPJ0S1	J0	R	R	R	R	R	R	R	R	R	R	R	R	R	S	R	R	R	R	S	NO	YES	YES	YES	NO	NO	YES	YES	YES	NO	YES	NO
P15KPJ10Su	J5	R	I	R	S	R	S	R	R	I	R	R	I	R	S	R	R	R	R	R	NO	YES	YES	YES	YES	NO	NO	NO	YES	YES	NO	NO
P16KPJ10Su	J5	I	R	R	S	R	R	R	S	R	R	R	R	R	S	R	R	R	R	S	NO	YES	YES	YES	YES	NO	NO	NO	YES	NO	YES	NO
P18KPJ5S2	J5	R	I	R	R	R	R	S	R	R	R	S	R	R	R	R	R	R	R	R	NO	YES	YES	YES	YES	NO	YES	YES	YES	YES	NO	NO
P26KPJ10Su	J5	I	R	R	R	R	R	R	R	R	R	R	R	R	R	R	R	R	R	R	YES	YES	YES	NO	NO	NO	YES	YES	YES	YES	NO	NO
P31KPJ0S1	J0	I	R	R	I	I	R	R	R	I	R	R	I	R	R	R	R	R	R	R	NO	YES	YES	YES	NO	NO	NO	NO	YES	YES	NO	NO
P35KPJ5S3	J5	R	R	R	S	R	R	I	R	R	R	R	R	R	R	R	R	R	R	R	NO	YES	YES	YES	NO	NO	YES	YES	YES	YES	NO	NO
P36KPJ10Su	J5	R	R	R	S	R	R	R	R	R	R	R	R	R	R	R	R	R	R	R	NO	YES	YES	YES	NO	NO	YES	YES	YES	YES	NO	NO
P37KPJ10S2	J5	R	R	R	S	R	R	I	R	R	I	R	R	R	R	R	S	R	R	S	YES	YES	YES	NO	NO	NO	NO	NO	YES	YES	NO	NO
P38KPJ5S2	J5	R	R	R	I	R	R	R	R	I	R	I	R	R	R	R	R	R	S	S	NO	YES	YES	YES	YES	NO	NO	NO	YES	YES	NO	NO
P39KPJ5S2	J5	R	R	R	S	R	R	R	R	R	R	R	S	S	I	R	R	R	R	S	NO	YES	YES	YES	YES	NO	NO	NO	YES	NO	YES	NO
P47KPJ10S2	J5	R	R	R	S	R	R	R	R	R	R	R	R	R	S	R	R	R	R	S	NO	YES	YES	YES	YES	NO	YES	YES	YES	NO	YES	NO
P52KPJ0S3	J0	I	R	R	R	R	R	R	R	R	I	R	R	I	S	R	R	R	S	S	NO	YES	YES	YES	NO	YES	YES	YES	YES	YES	NO	NO
P55KPJ10Su	J5	R	R	R	R	I	I	R	R	I	R	I	I	S	S	I	R	R	S	S	NO	YES	YES	YES	NO	NO	YES	YES	YES	NO	YES	NO
P56KPJ10Su	J5	R	R	R	R	R	R	R	R	R	R	R	R	R	R	R	R	S	R	S	NO	YES	YES	YES	NO	NO	YES	YES	YES	YES	NO	NO
P60KPJ10S1	J5	R	R	R	R	R	R	I	R	R	R	R	R	R	R	R	R	R	R	S	YES	YES	YES	NO	NO	NO	NO	NO	YES	YES	NO	NO
P61KPJ10S2	J5	R	R	R	R	R	R	R	R	R	R	R	R	R	R	R	R	R	R	S	NO	YES	YES	YES	NO	NO	NO	NO	YES	YES	NO	NO
P62KPJ0S2	J0	R	R	R	R	R	R	R	R	R	I	R	R	R	S	R	R	R	R	S	NO	YES	YES	YES	NO	NO	YES	YES	YES	YES	NO	NO
P63KPJ5S1	J5	I	R	R	S	R	R	R	R	R	R	R	R	R	S	R	R	I	R	R	NO	YES	YES	YES	NO	NO	YES	YES	YES	NO	YES	NO
P66KPJ5Su	J5	R	R	R	S	R	R	I	R	R	R	R	R	R	S	R	R	I	R	S	NO	YES	YES	YES	NO	NO	YES	YES	YES	YES	NO	NO
P69KPJ10S2	J5	R	R	R	R	R	R	R	R	R	R	R	R	R	R	R	R	R	R	R	NO	YES	YES	YES	YES	NO	YES	YES	YES	YES	NO	NO
P70KPJ10S1	J5	R	R	R	R	R	R	R	R	R	R	R	R	R	R	R	R	R	R	R	NO	YES	YES	NO	NO	NO	NO	NO	YES	YES	NO	NO
P75KPPJ10Su	J5	I	R	R	I	R	R	R	R	R	I	R	R	R	R	R	R	R	R	R	YES	YES	YES	YES	NO	NO	YES	YES	YES	YES	NO	NO
P80KPJ10S1	J5	I	R	R	I	R	R	R	R	R	R	R	R	R	R	R	R	R	R	R	NO	YES	YES	YES	NO	NO	YES	YES	YES	NO	YES	NO
P81KPJ0S1	J0	I	R	R	I	R	R	R	R	R	R	R	R	R	R	R	R	R	R	R	NO	YES	YES	YES	NO	NO	NO	NO	YES	YES	NO	NO
P88KPJ10Su	J5	R	R	R	I	R	R	R	R	R	R	R	R	R	R	R	S	R	R	S	NO	YES	YES	YES	NO	NO	NO	NO	YES	YES	NO	NO
P90KPJ10S2	J5	R	I	R	I	R	R	R	R	R	R	I	R	R	R	R	R	I	S	S	YES	YES	YES	YES	NO	NO	NO	NO	YES	YES	NO	NO
P92KPJ10Su	J5	I	R	R	S	R	R	R	R	R	R	R	S	S	I	R	R	R	R	S	NO	YES	YES	YES	NO	YES	YES	YES	YES	NO	YES	NO
P93KPJ5Su	J5	S	S	R	S	R	R	R	I	R	R	R	R	R	S	R	R	R	R	S	NO	YES	YES	YES	NO	NO	YES	YES	YES	YES	NO	NO
P95KPJ0Su	J0	S	S	R	S	R	R	R	R	R	R	R	R	R	S	R	R	R	R	R	NO	YES	YES	YES	NO	NO	YES	YES	YES	YES	NO	NO
P97KPJ10S1	J5	S	S	R	S	R	R	R	R	R	R	R	R	R	S	R	R	R	R	S	NO	YES	YES	YES	NO	NO	NO	NO	YES	YES	NO	NO
P98KPJ10Su	J5	S	S	R	S	R	R	R	R	R	R	R	R	R	S	R	R	R	R	S	NO	YES	YES	YES	YES	NO	YES	YES	YES	YES	NO	NO
P2KPJ5Su	J5	S	S	R	S	S	S	S	S	S	R	S	S	R	R	I	R	R	R	S	NO	YES	YES	YES	NO	NO	YES	YES	YES	NO	NO	YES
P5KPJ5S2	J5	S	S	R	S	S	S	S	S	S	S	S	S	R	R	I	R	R	R	S	NO	YES	YES	YES	NO	NO	NO	YES	YES	NO	NO	NO
P7KPJ5S2	J5	S	S	R	S	S	S	S	S	S	S	S	S	R	R	I	R	R	R	S	NO	YES	YES	YES	NO	NO	YES	YES	YES	NO	NO	NO
P9KPJ0S3	J0	S	S	R	S	S	S	I	S	S	S	I	S	R	R	I	R	R	R	S	NO	YES	YES	YES	NO	NO	YES	YES	YES	NO	NO	NO
P10KPJ0S1	J0	S	I	R	S	S	S	S	S	S	S	S	S	R	R	I	R	R	R	S	NO	YES	YES	YES	NO	NO	NO	NO	YES	NO	NO	NO
P12KPJ0Su	J0	I	S	R	S	S	S	S	S	S	S	S	S	R	R	I	R	R	R	S	NO	YES	YES	YES	NO	NO	YES	YES	YES	NO	NO	NO
P13KPJ5S2	J5	I	S	R	S	S	S	S	S	S	R	S	S	R	R	I	R	R	R	S	NO	YES	YES	YES	YES	NO	YES	YES	YES	NO	NO	NO
P14KPJ0S1	J0	I	S	R	S	S	S	S	S	S	R	S	S	R	R	I	R	R	R	S	NO	YES	YES	YES	NO	NO	NO	NO	YES	NO	NO	NO
P17KPJ5S2	J5	I	S	R	S	S	S	S	S	S	S	S	S	R	R	I	R	R	R	S	NO	YES	YES	YES	NO	NO	YES	YES	YES	NO	NO	NO
P19KPJ5Su	J5	I	I	R	S	S	S	S	S	S	S	S	S	R	R	I	R	R	R	S	NO	YES	YES	YES	NO	NO	YES	YES	YES	NO	NO	NO
P20KPJ5Su	J5	I	S	R	S	S	S	S	S	S	R	S	S	R	R	I	R	R	R	S	NO	YES	YES	YES	NO	YES	NO	YES	YES	NO	NO	NO
P21KPJ5S1	J5	I	S	R	S	S	S	S	S	S	S	S	S	R	R	I	R	R	R	S	NO	YES	YES	YES	NO	NO	YES	YES	YES	NO	NO	YES
P22KPJ0Su	J0	I	S	R	S	S	S	S	S	S	S	S	S	R	R	I	R	R	R	S	NO	YES	YES	YES	NO	NO	NO	NO	YES	NO	NO	NO
P23KPJ5S3	J5	I	I	R	R	S	S	S	S	S	R	S	S	R	R	I	R	R	R	S	NO	YES	YES	YES	NO	NO	YES	YES	YES	NO	NO	NO
P27KPJ0S1	J0	I	S	R	S	S	S	S	S	S	S	R	S	R	R	I	R	R	R	S	NO	YES	YES	YES	NO	NO	YES	YES	YES	NO	NO	NO
P28KPJ5Su	J5	R	S	R	S	S	S	S	S	S	S	S	S	R	R	I	R	R	R	S	NO	YES	YES	YES	NO	NO	NO	NO	YES	NO	NO	NO
P30KPJ5Su	J5	R	S	R	S	S	S	S	S	S	S	S	S	R	R	I	R	R	R	S	NO	YES	YES	YES	NO	NO	NO	NO	YES	NO	NO	NO
P32KPJ10S1	J5	R	I	R	S	S	S	S	S	S	R	S	S	R	R	I	R	R	R	S	NO	YES	YES	YES	NO	NO	NO	YES	YES	NO	NO	NO
P33KPJ10S2	J5	R	I	R	S	S	S	S	S	S	S	R	S	R	R	I	R	R	R	S	NO	YES	YES	YES	NO	YES	YES	YES	YES	NO	NO	NO
P34KPJ5S1	J5	I	S	R	S	S	S	S	S	S	S	S	S	R	R	I	R	R	R	S	NO	YES	YES	YES	NO	NO	YES	YES	YES	NO	NO	NO
P40KPJ5S3	J5	I	S	R	S	S	S	S	S	S	R	S	S	R	R	I	R	R	R	S	NO	YES	YES	YES	NO	NO	YES	YES	YES	NO	NO	NO
P42KPJ5Su	J5	I	I	R	S	S	S	S	S	S	R	S	S	R	R	I	R	R	R	S	NO	YES	YES	YES	NO	NO	NO	YES	YES	NO	NO	NO
P43KPJ5S2	J5	R	I	R	S	S	S	S	S	S	S	S	S	R	R	I	R	R	R	S	NO	YES	YES	YES	YES	YES	YES	YES	YES	NO	NO	NO
P44KPJ10Su	J5	R	S	R	S	S	S	S	S	S	S	S	S	R	R	I	R	R	R	S	NO	YES	YES	YES	NO	NO	YES	YES	YES	NO	NO	NO
P45KPJ0Su	J0	I	S	R	S	S	S	S	S	S	R	R	S	R	R	I	R	R	R	S	NO	YES	YES	YES	NO	NO	YES	YES	YES	NO	NO	NO
P46KPJ0S1	J0	R	S	R	S	S	S	S	S	S	S	S	S	R	R	I	R	R	R	S	NO	YES	YES	YES	NO	NO	NO	NO	YES	NO	NO	NO
P48KPJ5Su	J5	I	I	R	R	S	S	S	S	S	S	S	S	R	R	I	R	R	R	S	NO	YES	YES	YES	NO	NO	NO	NO	YES	NO	NO	NO
P49J0S2	J0	R	I	R	S	S	S	S	S	S	S	S	S	R	R	I	R	R	R	S	NO	YES	YES	YES	NO	NO	NO	NO	YES	NO	NO	NO
P50KPJ5Su	J5	R	S	R	S	S	S	S	S	S	R	R	S	R	R	I	R	R	R	S	NO	YES	YES	YES	NO	NO	NO	NO	YES	NO	NO	NO
P53KPJ5S1	J5	R	S	R	S	S	S	S	S	S	R	S	R	R	R	I	R	R	R	S	NO	YES	YES	YES	NO	NO	YES	YES	YES	NO	NO	NO
P54KPJ0S2	J0	I	I	R	S	S	S	S	S	S	S	S	S	R	R	I	R	R	R	S	NO	YES	YES	YES	NO	YES	YES	YES	YES	NO	NO	NO
P57KPJ5S1	J5	I	I	R	R	S	S	S	S	S	S	S	S	R	R	I	R	R	R	S	NO	YES	YES	YES	NO	YES	YES	YES	YES	NO	NO	NO
P58KPJ0Su	J0	I	S	R	S	S	S	S	S	S	S	S	R	R	R	I	R	R	R	S	NO	YES	YES	YES	NO	NO	YES	YES	YES	NO	NO	NO
P59KPJ0S1	J0	S	S	R	S	S	S	S	S	S	R	S	S	R	R	I	R	R	R	S	NO	YES	YES	YES	NO	NO	YES	YES	YES	NO	NO	NO
P64KPJ5S3	J5	I	S	R	S	S	S	S	S	S	S	R	S	R	R	I	R	R	R	S	NO	YES	YES	YES	NO	NO	NO	YES	YES	NO	NO	NO
P65KPJ10Su	J5	I	S	R	S	S	S	S	S	S	S	S	S	R	R	I	R	R	R	S	NO	YES	YES	YES	NO	NO	NO	NO	YES	NO	NO	NO
P68KPJ5Su	J5	S	I	R	S	S	S	S	S	S	R	S	S	R	R	I	R	R	R	S	NO	YES	YES	YES	NO	NO	YES	YES	YES	NO	NO	NO
P72KPJ0Su	J0	S	S	R	S	S	S	S	S	S	S	S	S	R	R	I	R	R	R	S	NO	YES	YES	YES	NO	NO	YES	YES	YES	NO	NO	NO
P73KPJ5Su	J5	S	S	R	S	S	S	S	S	S	R	R	R	R	R	I	R	R	R	S	NO	YES	YES	YES	NO	YES	YES	YES	YES	NO	NO	NO
P74KPJ10S2	J5	I	S	R	S	S	S	S	S	S	I	S	S	R	R	I	R	R	R	S	NO	YES	YES	YES	NO	NO	NO	NO	YES	NO	NO	NO
P76KPJ0Su	J0	I	S	R	S	S	S	S	S	S	S	S	S	R	R	I	R	R	R	S	NO	YES	YES	YES	NO	NO	YES	YES	YES	NO	NO	NO
P77KPJ0Su	J0	S	I	R	S	S	S	S	S	S	S	S	S	R	R	I	R	R	R	S	NO	YES	YES	YES	NO	NO	YES	YES	YES	NO	NO	NO
P78KPJ0S1	J0	I	I	R	S	S	S	S	S	S	S	S	S	R	R	I	R	R	R	S	NO	YES	YES	YES	NO	NO	NO	NO	YES	NO	NO	NO
P79KPJ5S1	J5	I	S	R	S	S	S	S	S	S	S	S	S	R	R	I	R	R	R	S	NO	YES	YES	YES	NO	NO	YES	YES	YES	NO	NO	NO
P82KPJ5S3	J5	S	S	R	S	S	S	S	S	S	I	S	S	R	R	I	R	R	R	S	NO	YES	YES	YES	NO	NO	YES	YES	YES	NO	NO	NO
P83KPJ5S2	J5	S	I	R	S	S	S	S	S	S	S	S	S	R	R	I	R	R	R	S	NO	YES	YES	YES	NO	NO	YES	YES	YES	NO	NO	NO
P84KPJ0S1	J0	I	S	R	S	S	S	S	S	S	S	S	S	R	R	I	R	R	R	S	NO	YES	YES	YES	NO	NO	NO	YES	YES	NO	NO	NO
P85KPJ5Su	J5	I	S	R	S	S	S	S	S	S	I	S	S	R	R	I	R	R	R	S	NO	YES	YES	YES	NO	NO	NO	NO	YES	NO	NO	NO
P86KPJ10Su	J5	I	S	R	S	S	S	S	S	S	S	S	S	R	R	I	R	R	R	S	NO	YES	YES	YES	NO	NO	YES	YES	YES	NO	NO	NO
P87KPJ0Su	J0	I	S	R	S	S	S	S	S	S	S	S	S	R	R	I	R	R	R	S	NO	YES	YES	YES	NO	NO	YES	YES	YES	NO	NO	NO
P89KPJ10S2	J5	S	S	R	S	S	S	S	S	S	S	S	S	R	R	I	R	R	R	S	NO	YES	YES	YES	NO	NO	YES	YES	YES	NO	NO	NO
P91KPJ10Su	J5	S	S	R	S	S	S	S	S	S	S	S	S	R	R	I	R	R	R	S	NO	YES	YES	YES	YES	YES	NO	NO	YES	NO	NO	NO
P94KPJ5S1	J5	I	I	R	S	S	S	S	S	S	S	S	S	R	R	I	R	R	R	S	NO	YES	YES	YES	NO	NO	YES	YES	YES	NO	NO	NO
P96KPJ0Su	J0	I	I	R	S	S	S	S	S	S	S	S	S	R	R	I	R	R	R	S	NO	YES	YES	YES	NO	NO	YES	YES	YES	NO	NO	NO
